# 448. COVID-19 Acute Care at Home: A Substitution for Hospitalization in Patients with Mild Symptoms

**DOI:** 10.1093/ofid/ofab466.647

**Published:** 2021-12-04

**Authors:** Joel A Kammeyer, Brian Perkins, Sara Seegert, Dave E Caris, Caitlyn M Hollingshead, Evan D Brochin, Benjamin H Russell

**Affiliations:** 1 University of Toledo College of Medicine and Life Sciences, Toledo, Ohio; 2 ProMedica-Toledo Hospital, Toledo, Ohio; 3 University of Toledo College of Medicine, Toledo, Ohio

## Abstract

**Background:**

Constraints on resources require healthcare systems to implement alternative and innovative means for delivering care. The COVID-19 pandemic amplified this issue throughout the world, leading to shortages of ventilators, hospital beds, and healthcare personnel. We report the results of an Acute Care at Home Program (ACHP) response to COVID-19, providing in-home hospital-level care to patients with mild symptoms, preserving in-hospital beds for more serious illness.

**Methods:**

Patients with COVID-19 were selected for ACHP after undergoing risk stratification for severe disease, including oxygen evaluation, time course of illness, and evaluation of comorbidities. Patients admitted to ACH met inpatient criteria, required oxygen supplementation of ≤4 liters, and received insurance approval. Services were provided consistent with best practice of inpatient care, including 24/7 provider availability via TeleMedicine, bedside care provided by paramedics and nurses, respiratory therapy, radiology and laboratory services, pulse oximetry monitoring, and administration of medications. Protocols existed for patient transfer to hospital in the event of clinical deterioration.

**Results:**

Our initial cohort included 62 patients enrolled October 1, 2020 – May 31, 2021. Of these, 57 patients were discharged successfully from ACHP. Patients presented with initial oxygen requirements of 0-4 liters. Average length-of-stay in ACHP was 5.4 days. Five patients required hospitalization after enrollment in ACHP; one subsequently expired, two were discharged home, one returned to ACHP after inpatient hospitalization, and one remains hospitalized. One additional patient that was successfully discharged home from ACHP was later readmitted and expired in a subsequent hospitalization. The patients that expired had significant immunocompromising conditions that may have contributed to their outcomes.

**Conclusion:**

ACHP can provide care equivalent to hospitalization for select COVID-19 patients. Immunocompromised hosts with COVID-19 may represent a subset of patients in which in-house hospitalization must be carefully considered, even with mild oxygen requirements. Health systems should consider ACHP as a substitution for hospitalization for COVID-19 patients with mild symptoms.

**Disclosures:**

**All Authors**: No reported disclosures

